# High levels of chemerin associated with variants in the *NOS3* and *APOB* genes in rural populations of Ouro Preto, Minas Gerais, Brazil

**DOI:** 10.1590/1414-431X20209113

**Published:** 2020-05-08

**Authors:** A.P. Batista, K.F. Barbosa, C.Z. Masioli, E.M. Queiroz, C.C. Marinho, A.P.C. Cândido, G.L.L. Machado-Coelho

**Affiliations:** 1 Núcleo de Pesquisa em Ciências Biológicas, Programa de Pós-Graduação em Ciências Biológicas, Instituto de Ciências Exatas e Biológicas, Universidade Federal de Ouro Preto, Ouro Preto, MG, Brasil; 2 Laboratório de Epidemiologia, Departamento de Medicina de Família, Saúde Mental e Coletiva, Escola de Medicina, Universidade Federal de Ouro Preto, Ouro Preto, MG, Brasil; 3 Programa de Pós-Graduação em Saúde e Nutrição, Escola de Nutrição, Universidade Federal de Ouro Preto, Ouro Preto, MG, Brasil; 4 Departamento de Clínica Médica, Faculdade de Medicina, Universidade Federal de Minas Gerais, Belo Horizonte, MG, Brasil; 5 Departamento de Nutrição, Instituto de Ciências Biológicas, Universidade Federal de Juiz de Fora, Juiz de Fora, MG, Brasil

**Keywords:** Genetic epidemiology, Adipokines, Dyslipidemia, Obesity, Diabetes, Hypertension

## Abstract

Chemerin is an adipokine that has been associated with components of metabolic syndrome. It has been described to affect adipocyte metabolism and inflammatory responses in adipose tissue, as well as the systemic metabolism of lipids and glucose. Few epidemiological studies have evaluated classical and genetics cardiovascular risk factors (CVRFs) in the mixed adult rural population in Brazil. Therefore, the present study explored possible associations between CVRFs and chemerin. This cross-sectional study included 508 adults from the rural localities of Lavras Novas, Chapada, and Santo Antônio do Salto in Ouro Preto, Minas Gerais, Southeast Brazil. Demographic, behavioral, clinical, biochemical, anthropometric variables, and 12 single nucleotide polymorphisms (SNPs) linked with metabolic syndrome phenotypes were evaluated for associations with chemerin level. There was a significant association of high triglyceride levels [odds ratio (OR)=1.91, 95%CI: 1.23−2.98], insulin resistance (OR=1.82, 95%CI: 1.03−3.22), age (OR=1.64, 95%CI: 1.08−2.49), and sex (OR=1.99, 95%CI: 1.35−2.95) with high levels of chemerin. High chemerin levels were significantly associated with the genetic polymorphisms rs693 in the *APOB* gene (OR=1.50, 95%CI: 1.03−2.19) and rs1799983 in the *NOS3* gene (OR=1.46, 95%CI: 1.01−2.12) for the AA and GT+TT genotypes, respectively. In the concomitant presence of genotypes AA of rs693 and GT+TT of rs1799983, the chance of presenting high levels of chemerin showed a 2.21-fold increase (95%CI: 1.25−3.88) compared to the reference genotype. The development of classical CVRFs in this population may be influenced by chemerin and by two risk genotypes characteristic of variants in well-studied genes for hypertension and dyslipidemia.

## Introduction

Over the last few decades, cardiovascular disease (CVD) has been recognized as one of the world’s leading causes of death (1). The burden of CVD can be projected in terms of risk factors, often portrayed as modifiable or non-modifiable (1).

In addition to the classical risk factors, new biomarkers of cardiovascular risk have been explored. Among them is chemerin, which is an adipokine also known as RARRES2 (retinoic acid receptor-responding protein 2). It was originally described as a chemoattractant for cells of the immune system (2). Since 2007, both chemerin and its receptor CMKLR1 have been found to be strongly expressed in white, visceral, and subcutaneous adipose tissue from animal models and in humans. Subsequently, chemerin was identified as a new adipokine with potential autocrine and paracrine functions (2,3). In recent years, several studies have discussed its role as a cardiovascular risk biomarker considering its association with components of metabolic syndrome, including obesity and overweight (MIM: 601665), type 2 diabetes mellitus (T2DM) (MIM: 125853), dyslipidemias (MIM: 143890), and arterial hypertension (AH) (MIM: 145500) (2,4,5).

In addition to clinical and biochemical cardiovascular risk factors (CVRFs), research has steadily improved the understanding of the genetic basis of complex human diseases. Remarkable advances have been made in the discovery of new genetic associations with classical CVRFs such as dyslipidemias, AH, T2DM, and obesity (6).

Ouro Preto, Minas Gerais (MG) is located in the southeast region of Brazil. Its population is formed by the miscegenation of Europeans, Africans, and Amerindians, historically gathered by the economic activity based on gold extraction. In 2001, the study “Hearts of Ouro Preto” described a disturbingly high prevalence of classical CVRFs such as AH, hyperglycemia, dyslipidemia, obesity, and central obesity in the adult urban population of Ouro Preto (7,8), as well as in children and adolescents (9), emphasizing that prompt action was required.

To date, few epidemiological studies have evaluated classical and emerging risk factors in the mixed adult rural population in Brazil. Therefore, the present study explored possible associations between classical CVRFs and chemerin. The objective was to evaluate it as a candidate predictor of cardiovascular risk in two rural localities of Ouro Preto. In addition, a panel of 12 single nucleotide polymorphisms (SNPs) was genotyped in genes previously described as modulators of cardiovascular risk, including the chemerin-encoding *RARRES2* gene, to evaluate whether chemerin is associated with genetic variants.

## Material and Methods

### Ethical aspects

This study was approved by the Human Research Ethics Committee of the Federal University of Ouro Preto (No. 125017/2015 and CAAE 51666115.5.0000.5150). All participants provided written consent after receiving broad information.

### Study design and population

A population-based cross-sectional study was carried out in two rural districts of Ouro Preto, namely Lavras Novas, including its subdistrict Chapada, and Santo Antônio do Salto, between May 2015 and December 2016. Men and women >18 years and living in the localities for at least 5 years were considered eligible. Adults who did not complete the questionnaire, did not attend the collection of blood samples, or refused to provide written informed consent were not included.

### Sample size and sampling process

A sample size of 600 subjects, including 10% losses, was defined based on the following assumptions: study population of 1509 subjects, confidence level of 95%, study power of 99%, expected frequency of AH in the population 38%, and expected error of 5%. The sampling process was based on stratification by sex and age group, where all eligible residents were invited to participate in the study. A total of 508 participants were included in the analysis.

**Figure 1:**
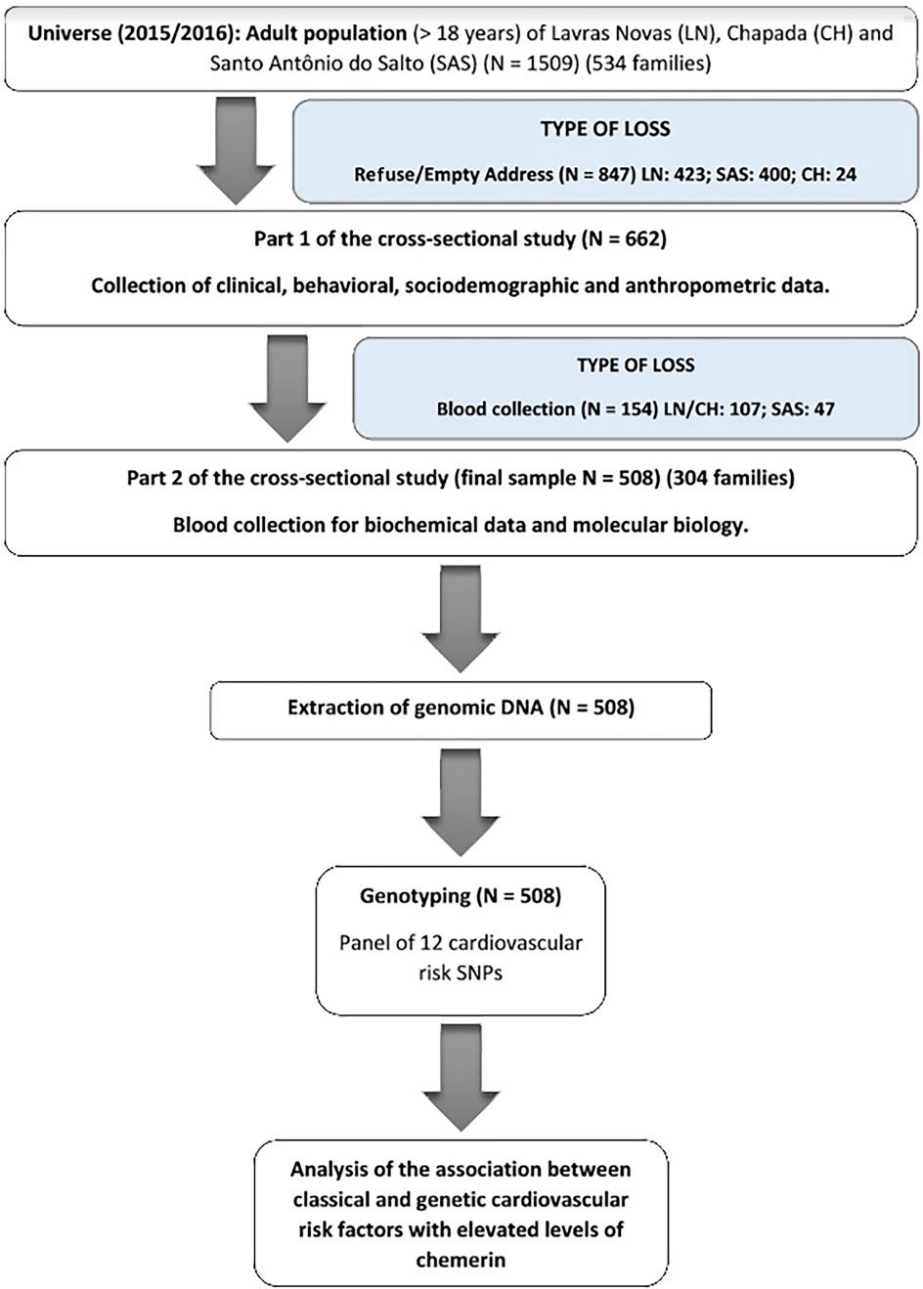
Sampling process and analysis stages of the present study. SNP: single nucleotide polymorphisms.

From a total of 1509 individuals eligible for the present study, 847 people refused consent or were absent from the home where the questionnaire was applied. Of the 662 who responded to the questionnaire, 154 did not attend the blood collection session, totaling 508 participants (Figure 1). The losses were non-differential by sex and greater in the elderly.

### Homogeneity of the sample in relation to losses

In order to observe the homogeneity of the sample regarding the representativeness of the population among the refusals, permission was requested to gauge the blood pressure, and after analysis, there was no significant difference between the participants and the refusal group (P=0.558). The mean systolic blood pressure (SBP) and diastolic blood pressure (DBP) in the participating group were 130.8 mmHg (±22.7) and 79.6 mmHg (±14.6), respectively, whereas in the refusal group, they were 133.1 mmHg (±16.0) and 83.8 mmHg (±12.6), respectively.

### Exposure, outcome, and confounders

Demographic, behavioral, clinical, biochemical, and anthropometric variables and 12 single nucleotide polymorphisms (SNPs) linked with metabolic syndrome phenotypes were evaluated as exposure. The levels of chemerin above the median were considered the outcome. Sex, age, and biochemical measurements were considered the main confounders.

### Sociodemographic and behavioral data

The sociodemographic characteristics (sex, age, income, marital status, education, and self-reported skin color), behavioral characteristics (smoking and alcohol consumption), and medication use were recorded in a semi-structured questionnaire filled in by trained interviewers. The classification of smoking status was based on self-reported past and/or current use of cigarettes. Three groups were considered: 1) never smokers: those who did not smoke or smoked less than 100 cigarettes throughout their lives; 2) ex-smokers: those who smoked at least 100 cigarettes during their lifetime but stopped smoking for more than 6 months; and 3) current smokers: those who had smoked 100 or more cigarettes during their lifetime and continued to smoke (10). For statistical analysis, current smokers and ex-smokers were grouped as ‘smokers’. The degree of dependence on alcohol drinking was evaluated using the Cut-down, Annoyed, Guilty, Eye-opener (CAGE) questionnaire (11).

### Risk factors for cardiovascular disease

The following classical CVRFs were evaluated independently: AH, T2DM, obesity, insulin resistance (IR), and dyslipidemia (data not shown).

#### Blood pressure

Three measures of blood pressure were taken at 1–2-min intervals with a HEM-705CP^®^ automatic digital blood pressure monitor (Omron Healthcare, USA). The participant was kept in a seated position, and the cuff was firmly fit and maintained at heart level. The lowest blood pressure measure was considered (12). Subjects who were on antihypertensives and/or had a casual measurement ≥140/90 mmHg were considered hypertensives (12). When SBP and DBP were in different categories, the highest category was used for classification.

#### Height and body weight

Stature was measured in centimeters (cm) with a portable stadiometer (Charder, Taiwan). The participant was requested to dress in light clothing and to stand barefoot with heels together in the center of the scale with his/her back to the marker, arms extended along the body, eyes fixed forward, erect head, and touching the vertical bar of the stadiometer (13). Body weight was measured with a TANITA^®^ (USA) portable scale with a maximum capacity of 150 kg and precision of 0.1 kg (13).

#### Body mass index (BMI)

BMI was calculated as weight (kg)/stature (m)^2^ and classified as ‘normal’ (<25 kg/m^2^), ‘overweight’ (25-29.9 kg/m^2^) and ‘obese’ (≥30 kg/m^2^) (14).

#### Waist circumference (WC)

WC was measured with a simple inelastic tape. The participants were requested to stand upright, abdomen relaxed, arms extended laterally along the body, feet slightly apart, and weight equally distributed between the legs. Cut-off points established for Brazilian adults were used for the classification of WC, taking cardiovascular risk as an outcome (15). Cut-off points at 87 cm for women and 95 cm for men were considered for cardiovascular risk.

### Blood biochemistry and molecular analysis

#### Lipid profile

The enzymatic-colorimetric method was used for the concentration of triglycerides (TG), total cholesterol, and high-density lipoprotein (HDL-c) fractions (Triglycerides Liquicolor Mono^®^, Cholesterol Liquicolor^®^, HDL Cholesterol Direct/Homogeneous Direct Test^®^, all Invitro Diagnostic/Human, Brazil; Automatic Analyzer Chemwell R6^®^, Awareness Technology, USA). Low-density lipoprotein (LDL-c) and very low-density lipoprotein (VLDL-c) fractions were calculated with Friedwald’s formula (1972) (16): LDL-c (mg/dL) = total cholesterol – HDL-c – (triglycerides/5) and VLDL-c(mg/dL) = triglycerides/5, when the triglyceride concentration was ≤400 mg/dL.

#### Definition of dyslipidemia

Dyslipidemias were classified according to the increased lipid fraction as follows: isolated hypercholesterolemia: isolated increase in LDL-c ≥160 mg/dL; isolated hypertriglyceridemia: isolated TG increase ≥150 mg/dL; mixed hyperlipidemia: increased LDL-c ≥160 mg/dL; TG ≥150 mg/dL; and HDL-c: reduction of HDL-c <40 mg/dL for men and <50 mg/dL for women, alone or in combination with increased LDL-c or TG (17). Dyslipidemia was defined as an increase in TG or LDL-c or total cholesterol ≥190 mg/dL, reduction in HDL-c, or any combination of the above.

#### Fasting glycemia

Fasting glycemia was measured by enzymatic colorimetric analysis without deproteinization (Enzyme Glucose, Invitro Diagnostic/Human, Brazil; Chemwell R6^®^ Automatic Analyzer, Awareness Technology, USA). Glycemia was classified as ‘normal’ (<100 mg/dL), ‘altered fasting glycemia’ (100–125.9 mg/dL), and ‘hyperglycemia’ (≥126 mg/dL) (18).

#### Definition of diabetes

Individuals on oral hypoglycemic medication or insulin and/or fasting glycemia ≥126 mg/dL were considered diabetic (18). Prediabetic subjects (100–125.9 mg/dL) who did not use hypoglycemic drugs were not considered diabetic.

#### Fasting insulinemia (FI)

FI was measured by indirect chemiluminescence according to the manufacturer’s protocol (Access 2 Immunoassay System^®^, Beckman Coulter, USA).

#### Insulin resistance

IR was determined by the Homeostatic Model Assessment of IR (HOMA-IR) index with the formula FI (μUI/mL) × fasting glycemia (mM) / 22.5 (19). The cut-off for IR in adults was 2.71 (20).

#### Chemerin

Blood levels of chemerin were measured with an enzyme-linked immunosorbent assay (ELISA) for human capture (Abcam Kit, Chemwell R6^®^ Automatic Analyzer, Awareness Technology, USA). The results were divided into medians for pooled analysis.

#### Extraction of genomic DNA

Genomic DNA extraction was performed with the Wizard^®^ Genomic DNA Purification Kit (Promega^®^, USA) according to the manufacturer’s protocol.

### Analysis of genetic markers of cardiovascular risk

The allelic and genotypic frequencies of a panel of 12 single nucleotide polymorphisms (SNPs) were evaluated and tested for Hardy-Weinberg equilibrium. The criteria for choosing the panel involved the following: 1) present a positive association with CVRFs (AH, obesity, dyslipidemias, T2DM, and IR) in at least five previous studies, with at least one being a Genome Wide Association Study (GWAS), and in ethnic groups similar to the study population; and 2) not present rare alleles in studies with African and European populations. The definition and characteristics of the SNPs are reported in Table 1 (5,21-27).

**Table 1:** Panel characteristics of 12 single nucleotide polymorphisms (SNPs) selected for the study.

Phenotype	RefSeqGene HGVS/refSNP	Chromosome	Gene/HGNC ID	Allele described in dbSNP	Reference
Hypertension	g.9543T>C/rs699	1q42.2	*AGT*/333	C/T (REV)	21
	g.12965T>G/rs1799983	7q36.1	*NOS3*/7876	G/T (FWD)	21
	g.10501C>T/rs5443	12p13.31	*GNB3*/4400	C/T (FWD)	21
Dyslipidemia	g.7903T>C/rs429358	19q13.32	*APOE*/613	C/T (FWD)	22
	g.8041C>T/rs7412	19q13.32	*APOE*/613	C/T (FWD)	22
	g.39751C>T/rs693	2p24.1	*APOB*/603	G/A (FWD)	23
	g.5912T>C/rs4520	11q23.3	*APOC3*/610	C/T (FWD)	24
	g.8017G>C/rs5128	11q23.3	*APOC3*/610	C/G (REV)	25
	g.35825T>C/rs5925	19p13.2	*LDLR*/6547	C/T (FWD)	26
Obesity, IR, and T2DM	g.68777C>G/rs1801282	3p25.2	*PPARG*/9236	C/G (FWD)	27
	c.515+281T>G/rs4721	7q36.1	*RARRES2*/9868	A/C (REV)	5
	c.280-494T>G/rs17173608	7q36.1	*RARRES2*/9868	G/T (FWD)	5

IR: insulin resistance; T2DM: type 2 diabetes mellitus; HGVS: Human Genome Variation Society; HGNC: HUGO Gene Nomenclature Committee.

Regarding the SNPs rs429358 and rs7412 in the *APOE* gene, there is the peculiarity that the combination of these characterizes a haplotype that generates three common variants called ε2, ε3, and ε4 (28). Also the combination of the alleles *APOE* rs429358 and rs7412 identify the isoforms: ε2 or APOE *2, ε3 or APOE *3, ε4 or APOE *4.

The allelic discrimination technique was performed by real-time PCR (qPCR) using a set of primers and probes specific for each SNP (TaqMan^®^ Minor Groove Binder-MGB, TaqMan^®^ System; 7500 Fast Real-Time PCR Systems, Applied Biosystems, USA).

### Statistical analysis

The database was constructed and analyzed using SPSS software version 22 (IBM, USA). The prevalence was calculated by the ratio between the number of cases for each CVRF and the total number of individuals in the sample. The allele frequencies were obtained by gene counting and tested for Hardy-Weinberg equilibrium (with the exception of the SNPs rs429358 and rs7412 *APOE* gene). Continuous variables were tested for normality of distribution by the Shapiro-Wilk test. For those with a normal distribution, one-way analysis of variance (ANOVA) was performed. Categorical variables were analyzed using Pearson’s chi-squared or Fisher’s exact test. The distribution of panel genotypes of 12 single nucleotide polymorphisms (SNPs) between the groups of chemerin quartiles was tested with Pearson’s chi-squared test. The binary logistic regression analysis was used to adjust associations for confounders (P<0.20), and the odds ratio (OR) was calculated to identify the genetic and classical markers associated with high levels of chemerin in a significant (P<0.05) and independent way.

## Results

A total of 329 women (64.8%, mean age 49.2±17.3) and 179 men (35.2%, mean age 49.9±16.6) participated. The age range was 18–91 years. Twenty participants (3.9%) lived in Chapada, 189 (36.7%) lived in Lavras Novas, and 306 (59.4%) lived in Santo Antônio do Salto. Table 2 shows the sociodemographic and behavioral characteristics of the study population and the levels of chemerin grouped in quartiles. Elderly, female, and illiterate groups were more likely to present higher levels of chemerin. Regarding marital status, higher levels of chemerin were found in married or in stable unions, followed by widowed and divorced participants.

**Table 2:** Socio-demographic and behavioral characteristics of the study population by quartiles of chemerin.

Variables	Chemerin (ng/mL)			OR (95%CI)	P*
	<160 (n=255)	≥160 (n=253)	Total (n=508)		
Age group					
18-34 years	84 (32.9)	67 (26.5)	151 (29.7)	1.0	
35-59 years	113 (44.3)	104 (41.1)	217 (42.7)	1.15 (0.76-1.75)	0.501
≥60 years	58 (22.8)	82 (32.4)	140 (27.6)	1.77 (1.11-2.82)	0.015
Gender					
Male	111 (43.5)	68 (26.9)	179 (35.2)	1.0	
Female	144 (56.5)	185 (73.1)	329 (64.8)	2.09 (1.44-3.04)	<0.0001
Schooling					
Literate	241 (94.5)	224 (88,9)	465 (91.7)	1.0	
Illiterate	14 (5.5)	28 (11.1)	42 (8.3)	2.15 (1.10-4.19)	0.022
Missing data**	0	1	1		
Marital status					
Not married	84 (32,9)	51 (20.2)	135 (26.6)	1.0	
Married or SU	146 (57.3)	163 (64.7)	309 (60.9)	1.85 (1.22-2.8)	0.003
Others^a^	25 (9.8)	38 (15.1)	63 (12.4)	2.5 (1.35-4.62)	0.002
Missing data**	0	1	1		
Income					
≥4 MS	27 (11.1)	40 (16.6)	67 (13.8)	1.0	
2 a 3 MS	147 (60.5)	122 (50.6)	269 (55.6)	0.56 (0.32-0.96)	0.035
≤1 MS	69 (28.4)	79 (32.8)	148 (30.6)	0.77 (0.42-1.38)	0.387
Missing data**	12	12	24		
Self-reported skin color					
White + yellow	31 (12.4)	31 (12.3)	62 (12.3)	1.0	
Black + brown	220 (87.6)	221 (87.7)	441 (87.7)	1.0 (0.59-1.71)	0.987
Missing data**	4	1	5		
Smoking^b^					
No	158 (62.7)	162 (64.8)	320 (63.7)	1.0	
Yes	94 (37.3)	88 (35.2)	182 (36.3)	0.91 (0.63-1.31)	0.642
Missing data**	3	3	6		
Alcohol drinking^c^					
Low risk/no alcohol drinking	227 (89.7)	226 (93.4)	453 (91.5)	1.0	
Alcohol dependence	26 (10.3)	16 (6.6)	42 (8.5)	0.61 (0.32-1.18)	0.144
Missing data**	2	11	13		

Data are reported as number and percent. SU: stable union; MS: minimum salary. *P<0.05 (Pearson’s chi-square test). **Missing data were excluded from analysis; ^a^separated/divorced or widowed; ^b^self-reported lifetime and/or current smoking; ^c^according to the CAGE (Cut-down, Annoyed, Guilty, Eye-opener) questionnaire.

The anthropometric, biochemical, and clinical characteristics according to quartiles of chemerin levels are shown in Table 3. There was a significant difference in chemerin levels between individuals with central obesity, high TG, and dyslipidemia than the reference group. In addition, altered glycemia, insulin levels above p75, and IR were associated with higher levels of chemerin.

**Table 3:** Anthropometric, clinical, and biochemical characteristics of the study population by quartiles of chemerin.

Variables	Chemerin (ng/mL)			OR (95%CI)	P*
	<160 (n=255)	≥160 (n=253)	Total (n=508)		
BMI (kg/m^2^)					
<25	123 (48.6)	101 (40.1)	224 (44.4)	1.0	0.054
≥25	130 (51.4)	151 (59.9)	281 (55.6)	1.45 (0.99-2.01)	
Missing data**	2	1	3		
WC (cm)					
Normal	157 (62.3)	120 (48.2)	277 (55.3)	1.0	0.001
Cardiovascular risk	95 (37.7)	129 (51.8)	224 (44.7)	1.77 (1.24-2.53)	
Missing data^**^	2	5	7		
TG (mg/dL)					
<150	208 (81.6)	171 (67.6)	379 (74.6)	1.0	<0.0001
≥150	47 (18.4)	82 (32.4)	129 (25.4)	2.12 (1.40-3.2)	
TC (mg/dL)					
<190	141 (55.3)	120 (47.4)	261 (51.4)	1.0	0.076
≥190	114 (44.7)	133 (52.6)	247 (48.6)	1.37 (0.96-1.94)	
LDL-c (mg/dL)					
<130	207 (81.2)	201 (79.4)	408 (80.3)	1.0	0.624
≥130	48 (18.8)	52 (20.6)	100 (19.7)	1.11 (0.72-1.72)	
HDL-c (mg/dL)					
≥40	231 (90.6)	225 (88.9)	456 (89.8)	1.0	0.0538
<40	24 (9.4)	28 (11.1)	52 (10.2)	1.19 (0.67-2.12)	
Dyslipidemia					
No	184 (72.2)	151 (59.7)	335 (65.9)	1.0	0.003
Yes	71 (27.8)	102 (40.3)	173 (34.1)	1.75 (1.20-2.53)	
FG (mg/dL)					
<100	136 (53.3)	102 (40.3)	238 (46.9)	1.0	
100-125	107 (42.0)	123 (48.6)	230 (45.2)	1.53 (1.06-2.20)	0.021
>125	12 (4.7)	28 (11.1)	40 (7.9)	3.11 (1.50-6.41)	0.001
FI (μLU/mL)					
<p75	207 (81.2)	174 (68.8)	381 (75.0)	1.0	0.001
≥p75	48 (18.8)	79 (31.2)	127 (25.0)	1.95 (1.29-2.95)	
HOMA-IR					
<2.71	231 (90.6)	203 (80.2)	434 (85.4)	1.0	0.001
≥2.71	24 (9,4)	50 (19.8)	74 (14.6)	2.37 (1.40-3.95)	
Diabetes					
No	239 (93.7)	220 (87.0)	459 (90.4)	1.0	0.01
Yes	16 (6.3)	33 (13.0)	49 (9.6)	2.24 (1.20-4.18)	
Hypertension					
No	151 (59.2)	140 (55.3)	291 (57.3)	1.0	0.377
Yes	104 (40.8)	113 (44.7)	217 (42.7)	1.17 (0.82-1.66)	
SBP (mmHg)					
Normal/borderline	165 (64.7)	164 (64.8)	329 (64.8)	1.0	0.978
High	90 (35.3)	89 (35.2)	179 (35.2)	0.99 (0.69-1.43)	
DBP (mmHg)					
Normal/borderline	206 (80.8)	204 (80.6)	410 (80.7)	1.0	0.965
High	49 (19.2)	49 (19.4)	98 (19.3)	1.01 (0.65-1.56)	

Data are reported as number and percent. *P<0.05 (Pearson’s chi-squared test). **Missing data were excluded from analysis; BMI: body mass index; WC: waist circumference; TG: triglycerides; TC: total cholesterol; LDL-c: low-density lipoprotein; HDL-c: high-density lipoprotein; FG: fasting glycemia; FI: fasting insulinemia; HOMA-IR: Homeostatic Model Assessment of Insulin Resistance; SBP: systolic blood pressure; DBP: diastolic blood pressure.

Table 4 presents the analysis of the allelic and genotypic frequencies of a panel of 12 genetic markers of the SNP type by quartiles of chemerin level. Levels were significantly higher in the presence of the GT genotype rs1799983 (g.12965T>G) of the *NOS3* gene and the AA genotype rs693 (g.39751C>T) polymorphism of the *APOB* gene compared to the respective ancestral homozygotes (GG for both).

**Table 4:** Frequency of genotypes of a panel of 12 single nucleotide polymorphisms (SNP)-like polymorphisms in the study population by quartiles of chemerin.

Gene/dbSNP rs ID	Genotypes^*^	Chemerin (ng/mL)			OR (95%CI)	P**
		<160 (n=255)	≥160 (n=253)	Total (n=508)		
*PPARG*/rs1801282	CC	248 (97.3)	247 (97.6)	495 (97.4)	1.0	
	CG	7 (2.7)	6 (2.4)	13 (2.6)	0.86 (0.28-2.59)	0.790
	GG	-	-	-		
*RARRES2*/rs4721	GG	61 (23.9)	49 (19. 3)	110 (21.7)	1.0	
	GT	116 (45.5)	134 (53.0)	250 (49.2)	1.43 (0.91-2.25)	0.113
	TT	78 (30.6)	70 (27.7)	148 (29.1)	1.11 (0.68-1.83)	0.661
*RARRES2*/rs17173608	TT	181 (71.0)	183 (72.3)	364 (71.7)	1.0	
	GT	62 (24.3)	63 (24.9)	125 (24.6)	1.00 (0.66-1.50)	0.980
	GG	12 (4.7)	7 (2.8)	19 (3.7)	0.57 (0.22-1.49)	0.253
*NOS3*/rs1799983	GG	144 (56.5)	120 (47.5)	264 (52.0)	1.0	
	GT	89 (34.9)	119 (47.0)	208 (40.9)	1.60 (1.11-2.31)	0.011
	TT	22 (8.6)	14 (5.5)	36 (7.1)	0.76 (0.37-1.55)	0.457
*GNB3*/rs5443	CC	27 (10.6)	34 (13.5)	61 (12.0)	1.0	
	CT	128 (50.2)	124 (49.0)	252 (49.6)	0.76 (0.43-1.35)	0.359
	TT	100 (39.2)	95 (37.5)	195 (38.4)	0.75 (0.42-1.34)	0.338
*APOB*/rs693	GG	142 (55.7)	161 (63.6)	303 (59.6)	1.0	
	AG	89 (34.9)	79 (31.2)	168 (33.1)	1.27 (0.87-1.86)	0.203
	AA	24 (9.4)	13 (5.2)	37 (7.3)	2.09 (1.02-4.26)	0.038
Alleles *APOE*/ rs429358 + rs7412	ε2	1 (0.4)	3 (1.2)	4 (0.8)	1.0	
	ε3	207 (81.2)	180 (71.1)	387 (76.2)	0.29 (0.01-2.75)	0.256
	ε4	47 (18.4)	70 (27.7)	117 (23.0)	0.49 (0.02 -4.82)	0.541
Genotypes *APOE*/ rs429358 + rs7412	E2/2	1 (0.4)	3 (1.2)	4 (0.8)	1.0	
	E2/3	23 (9.0)	14 (5.5)	37 (7.3)	0.20 (0.01-2.14)	0.151
	E2/4	184 (72.2)	166 (65.6)	350 (68.9)	0.30 (0.03-2.91)	0.273
	E3/3	5 (2.0)	5 (2.0)	10 (2.0)	0.33 (0.02-4.40)	0.393
	E3/4	40 (15.6)	59 (23.3)	99 (19.5)	0.49 (0.04-4.89)	0.537
	E4/4	2 (0.8)	6 (2.4)	8 (1.6)	1.0 (0.06-15.9)	0.999
*LDLR*/rs5925	CC	17 (6.7)	15 (5.9)	32 (6.3)	1.0	
	CT	73 (28.6)	68 (26.9)	141 (27.8)	0.94 (0.43-2.04)	0.890
	TT	165 (64.7)	170 (67.2)	335 (65.9)	0.85 (0.41-1.77)	0.675
*AGT*/rs699	GG	113 (44.4)	113 (44.7)	226 (44.5)	1.0	
	AG	110 (43.1)	119 (47.0)	229 (45.1)	0.92 (0.64-1.33)	0.675
	AA	32 (12.5)	21 (8.3)	53 (10.4)	1.52 (0.82-2.80)	0.173
*APOC3*/rs5128	GG	7 (2.7)	5 (2.0)	12 (2.4)	1.0	
	CG	41 (16.1)	43 (17.0)	84 (16.5)	0.68 (0.20-2.31)	0.537
	CC	207 (81.2)	205 (81.0)	412 (81.1)	0.72 (0.22-2.30)	0.581
*APOC3*/rs4520	CC	130 (51.0)	132 (52.2)	262 (51.6)	1.0	
	CT	93 (36.5)	99 (39.1)	192 (37.8)	0.95 (0.65-1.38)	0.803
	TT	32 (12.5)	22 (8.7)	54 (10.6)	1.47 (0.81-2.67)	0.196

*The ancestral allele was taken for the homozygous reference group with the exception of the APOE haplotype rs429358 and rs7412 where the reference was the ε2 allele. **P<0.05 (Pearson’s chi-squared test).

The multivariate analysis was performed by binary logistic regression adjusting the levels of chemerin by the anthropometric, body composition, biochemical, sociodemographic, behavioral, and genetic variables. Table 5 shows the variables that remained significant after adjustment.

**Table 5:** Binary logistic regression analysis of risk factors of cardiovascular disease associated with high levels of chemerin in the study population.

Variables	Crude OR (95%CI)	P	Adjusted OR^*^ (95%CI)	P
Gender				
Female	2.09 (1.44-3.04)	<0.0001	1.99 (1.35-2.95)	0.001
Age group				
Elderly	1.62 (1.09-2.41)	0.015	1.64 (1.08-2.49)	0.019
HOMA-IR				
Insulin resistance	2.37 (1.40-3.95)	0.001	1.82 (1.03-3.22)	0.038
TG				
≥150 mg/dL	2.12 (1.40-3.2)	<0.0001	1.91 (1.23-2.98)	0.004
rs1799983^a^				
GT+TT	1.43 (1.01-2.03)	0.041	1.46 (1.01-2.12)	0.043
rs693^b^				
AA	1.39 (0.97-1.98)	0.068	1.50 (1.03-2.19)	0.034

*Adjusted for sex, age, HOMA-IR (Homeostatic Model Assessment of Insulin Resistance), TG (triglycerides), and high-density lipoprotein cholesterol; ^a^reference group: genotype homozygous for the ancestral allele; ^b^risk group: genotype homozygous for the variant allele.

Table 6 shows the analysis of risk adjusted for sex, age, HOMA-IR, TG, and HDL-c for the chemerin groups in relation to the marker genotypes rs693 and rs1799983. Risk genotypes were not independently associated with high levels of chemerin. However, in the concomitant presence of the AA and GT+TT genotypes of polymorphisms rs693 and rs1799983, respectively, there was a 2.21-fold increase in the chance of elevated levels of chemerin (95%CI: 1.25−3.88) compared to the reference genotype (GG).

**Table 6:** Bivariate risk association of genotypes of the polymorphisms *APOB* rs693 and *NOS3* rs1799983 and levels of chemerin by quartiles in the study population.

Genotypes^*^		Chemerin (ng/mL)		Crude OR (95%CI)	P	Adjusted OR** (95%CI)	P
rs693	rs1799983	<160 (n, %)	≥160 (n, %)				
GG+GA	GG	56 (59.6)	38 (40.4)	1.0		1.0	
GG+GA	GT+TT	57 (51.4)	54 (48.6)	1.39 (0.80−2.43)	0.238	1.49 (0.84−2.67)	0.172
AA	GG	88 (51.8)	82 (48.2)	1.37 (0.82−2.28)	0.222	1.53 (0.89−2.61)	0.120
AA	GT+TT	54 (40.6)	79 (59.4)	2.15 (1.25−3.69)	0.005	2.21 (1.25−3.88)	0.006

*Reference genotype: rs693, GG+GA; rs1799983, GG; risk genotype: rs693, AA; rs1799983, GT+TT. **Adjusted for sex, age, Homeostatic Model Assessment of Insulin Resistance, triglycerides, and high-density lipoprotein cholesterol.

## Discussion

The present study evaluated adipokine chemerin according to classical and genetic CVRFs in rural populations of Ouro Preto, Brazil. There was a significant association of classical CVRFs with high levels of chemerin. Associations with high levels of TG and IR were significant after adjustment for confounders. The association of high levels of chemerin was also noted with the genetic polymorphisms rs693 in the *APOB* gene and rs1799983 in the *NOS3* gene for the AA and GT genotypes, respectively. The risk-modifying model showed that the concomitant presence of genotypes AA of rs693 and GT+TT of rs1799983 produced a 2.21-fold increase in the chance of presenting high levels of chemerin (95% CI: 1.25−3.88) compared to the reference genotype (GG).

The study by Bozaoglu et al. (2) has uncovered interesting aspects about chemerin, such as its association with metabolic syndrome phenotypes, obesity, AH, dyslipidemia, and hyperglycemia. Other studies also confirmed these findings (3,29). Interestingly, the average level of chemerin observed in the present study (203.9 ng/mL) was above the range considered physiological (70−150 ng/mL) (30). This result raised the question of whether the increased levels of chemerin in this population influenced the development of CVRFs.

In the present study, high triglyceride levels were significantly associated with high chemerin levels after risk-adjusted analysis (OR=1.91, 95% CI: 1.23−2.98). A strong positive correlation has been described between elevated levels of chemerin and dyslipidemia, and each component alone (3,5,31). Since dyslipidemia is frequently associated with obesity and overweight, the relationship of chemerin with this metabolic disorder could be explained by mechanisms that are interconnected. A possible path would be the potential action of chemerin on lipid metabolism in the liver, skeletal muscle, and adipose tissue and the stimulation of lipolysis in adipocytes (31). Nevertheless, Becker et al. (32) did not observe alterations in the lipid profile in animal models with increased expression of chemerin. Further investigation of the influence of chemerin on lipid homeostasis is needed.

T2DM and IR were associated with high levels of chemerin in the present study. The relationship between T2DM and chemerin has not been fully elucidated, but our findings were in agreement with several observational studies (3,31,33). Bobbert et al. (33) reported that chemerin is likely involved in the pathophysiology of T2DM. In fact, changes in chemerin levels may precede the onset of T2DM. It has been reported that chemerin regulates glucose metabolism through adipocytes in animal models and in humans (2,3).

Deletion of the chemerin receptor gene (CMKLR1) showed a protective effect against obesity and decreased insulin secretion by reducing glucose uptake in skeletal muscle and adipose tissue (34). The administration of exogenous chemerin decreased tissue glucose uptake and reduced serum levels of insulin in diabetic and obese murine models, whereas glucose uptake and insulin levels did not change with exogenous chemerin in non-obese and normoglycemic models (3). Therefore, our results corroborated the mechanism proposed by other studies, reinforcing a potential association of chemerin with disorders of glucose metabolism and IR and a possible link between them and obesity. Other experimental studies should be conducted to confirm these associations.

The present study is among the pioneers showing the association between the presence of variants within the *NOS3* gene (rs1799983) and the *APOB* gene (rs693) and elevated levels of chemerin in a mixed population in Brazil. Regarding the effects of variations in the *NOS3* gene, studies indicate that decreased bioavailability of NO is also involved in the pathophysiology of metabolic diseases, such as T2DM and obesity. Therefore, the contribution of *NOS3* gene polymorphisms, particularly the T allele of polymorphism rs1799983, to susceptibility to those conditions has been investigated (35). Interestingly, among African and Euro-American youth, carriers of the T allele of the same polymorphism had a higher BMI and WC (36). Another study reported that in addition to the T allele (OR=1.72; P=0.001), the presence of the TT genotype in northern Africans showed a nearly three-fold increased risk of developing obesity (OR=2.93; P=0.03) (37), suggesting that this polymorphism may influence genetic susceptibility to obesity. Evidence from the Genome-wide Association Study (GWAS) points to an association of the *APOB* gene and its polymorphism rs693 with changes in serum lipid levels (23). However, further investigation is required to elucidate whether this variation predisposes individuals to obesity, especially in mixed populations.

Taken together, our results provided evidence supporting the role of the variants rs1799983 in *NOS3* and rs693 in the *APOB* gene as modulators of chemerin through the increase in adiposity. Along these lines, increased adiposity stimulated chemerin levels to increase, which in turn induced further growth of adiposity in a positive feedback loop. More specifically, the interaction between the variants rs1799983 and rs693 may be linked to atherosclerosis and CVD by the adipokine chemerin.

The strength of this study was that it evaluated classical and emerging CVRFs in mixed rural populations, whereas few studies have investigated Brazilian populations. The sample was representative, making it possible to estimate the prevalence of the risk factors studied and ensure the external validity of the study. Nevertheless, the study had limitations. First, the cross-sectional design adopted did not allow any causal inference to be made between the factors analyzed and chemerin. Second, the sample size may have limited the results related to genetic polymorphisms. Further studies with larger samples are necessary to confirm the associations found.

In conclusion, our results indicated a high prevalence of classical CVRFs such as T2DM, overweight/obesity, dyslipidemia, and AH in rural populations of Ouro Preto, as well as high serum levels of the adipokine chemerin. We suggest that the development of CVRFs in this population may be influenced by two risk genotypes characteristic of variants in well-studied genes for hypertension (rs1799983) and dyslipidemia (rs693) and by chemerin as well.
